# Evaluation of the psychometric properties of the Reproductive Autonomy Scale for use in the UK

**DOI:** 10.1136/bmjsrh-2022-201685

**Published:** 2023-01-19

**Authors:** Eleanor Riches, Geraldine Barrett, Jennifer Anne Hall

**Affiliations:** EGA Institute for Women's Health, UCL, London, UK

**Keywords:** family planning services, patient advocacy, reproductive behavior, reproductive health services, surveys and questionnaires, contraception behavior

## Abstract

**Background:**

Reproductive autonomy—control over outcomes including contraceptive use and childbearing—is a human right and vital to women’s empowerment. Those whose reproductive autonomy is threatened by the structures and relationships in their lives are at risk of coercion and unplanned pregnancy and could benefit from additional services. The Reproductive Autonomy Scale (RAS) was developed in the USA to assess women’s reproductive autonomy; this study evaluates the RAS for use in the UK.

**Methods:**

After testing, the RAS was incorporated into an online survey of women of reproductive age. Those who were sexually active were asked to complete the RAS, which was evaluated according to classical test theory. Reliability was assessed via internal consistency and a 3-month test-retest. Construct validity was assessed using hypothesis testing and confirmatory factor analysis.

**Results:**

For 826 women the RAS was highly acceptable, with a response rate of >97.7%. Almost the whole range of reproductive autonomy scores were captured. Internal consistency was good, with a Cronbach’s α of 0.75. Test-retest reliability was fair-good with an intraclass correlation coefficient of 0.67. Construct validity analysis found the scale to be valid based on our hypothesis that among women who want to avoid pregnancy, those with higher reproductive autonomy will be more likely to use contraception. The three-factor structure of the scale was confirmed on confirmatory factor analysis.

**Conclusion:**

The RAS is valid and reliable for use in the UK. This tool holds potential utility across research, clinical practice, health interventions and policy development.

**WHAT IS ALREADY KNOWN ON THIS TOPIC**

- Reproductive autonomy is the ability to control outcomes such as contraceptive use and childbearing. It is a human right and a vital component of women’s empowerment.

**WHAT THIS STUDY ADDS**

- The Reproductive Autonomy Scale (RAS) is valid for use in the UK.

**HOW THIS STUDY MIGHT AFFECT RESEARCH, POLICY OR PRACTICE**

- A validated UK version of the RAS has potential for use in research, practice and policy by providing an accurate, multidimensional measure of women’s reproductive autonomy.

## Introduction

Reproductive autonomy is a human right. It is the ability to choose whether and when to have sex, become pregnant, use contraception, or continue a pregnancy.^1^ These decisions are also referred to as reproductive intentions and are an important component of women’s empowerment. The overall construct of women’s empowerment is complex and multidimensional, covering numerous domains—including the economic, sociocultural, interpersonal, legal-political, psychological and more.^2^ Women can be more empowered in certain aspects of their lives than others.^3^ With this in mind, a generalised measure of autonomy may not encompass a woman’s ability to achieve her reproductive intentions. Existing validated scales tend to focus on levels of autonomy associated with sexual activity rather than reproductive outcomes.^4–6^ Furthermore, interpersonal power in the reproductive domain is continuously influenced by a woman’s relationship with her partner or family, as well as the culture and context in which she lives. As these factors change, a woman’s level of reproductive autonomy will fluctuate.^7^

The Reproductive Autonomy Scale (RAS) was developed and evaluated for use in the USA in 2014.^7^ The authors found that women’s reproductive autonomy is a multidimensional construct, comprising three key factors. The final RAS comprised 14 items organised into three subscales; Decision Making (four items), Freedom from Coercion (five items), and Communication (five items). A score is produced for each subscale, measuring women’s level of reproductive autonomy in each area.

The RAS is unique and valuable because it is the first psychometric tool to measure specifically women’s ability to achieve their reproductive intentions. The RAS has been evaluated in Vietnam^8^ and Brazil.^9^ While found to be valid in Brazil, the evaluation in Vietnam raised questions about its validity in this setting. This is likely because the Vietnam sample did not represent the full spectrum of the construct of reproductive autonomy. This study aims to evaluate the RAS for use in the UK. A validated UK version of the RAS would be a useful tool across research, clinical practice and health interventions.

## Methods

A cohort of non-pregnant women were recruited in the UK in October 2018 via social media adverts (Facebook and Instagram), posters and word of mouth. The survey screening questions asked participants whether they were female, aged 15 or over, had not gone through menopause or had been sterilised. The target sample size was 1000 participants, which is considered ‘excellent’ for validation studies.^10^ Women self-completed an online survey, using RedCap,^11 12^ containing the RAS and also covering obstetric history, pregnancy preferences for the future, contraceptive use or pregnancy preparation, and sociodemographics. The RAS was subjected to cognitive interviews^13^ to check women’s understanding of the questions and responses before recruitment commenced. Only women who confirmed they had engaged in vaginal sex with a male partner in the last year were asked to participate in the RAS. For the evaluation of the RAS, we used survey data completed at baseline and 3 months.

A Classical Test Theory-based approach to analysis was used, in keeping with the original US development study. Rates of missing data were assessed to indicate the scale’s level of acceptability.^14^ To assess item discrimination and range of responses, the item endorsement values were examined to see if any item response category had an endorsement >80%.^15^ The distribution of scores (of those with full data) for total reproductive autonomy and each subscale were analysed to see if the full range of scores were present and to evaluate the targeting of the scale.

Test-retest reliability was analysed by calculating the intraclass correlation coefficient between the baseline total average reproductive autonomy scores and at the 3-month interval, with 0.5–0.75 indicating moderate reliability, 0.76–0.9 indicating good reliability, and above 0.9 as almost perfect reliability.^16^ Internal consistency was evaluated by calculating Cronbach’s α, with accepted criteria of 0.7 indicating good reliability.^17^ Cronbach’s α was calculated for the overall scale, as well as each of the three subscales.

Construct validity was examined using hypothesis testing. The hypothesis tested was: ‘Among women who want to avoid pregnancy, those with higher reproductive autonomy will have greater odds of using contraception’. Contraceptive use was defined as having used any method of contraception in the last 30 days, including vasectomy, natural family planning, withdrawal, breastfeeding and the emergency pill. This hypothesis was tested using the Kruskal-Wallis test and by multivariable logistic regression, adjusting for age, relationship status, ethnicity, education and employment.

As the RAS was shown to have three factors during its development, we examined structural validity, also an aspect of construct validity, by using confirmatory factor analysis to compare the three-factor solution to a one-factor solution, and to check that the items loaded onto each factor as expected. To confirm the data were suitable for factor analysis, we assessed the polychoric correlation matrix, Bartlett test of sphericity and Kaiser-Meyer-Olkin (KMO) measure of sampling adequacy; KMO values between 0.8 and 1 indicate the sampling is adequate.^17^ The Akaike’s (AIC) and Bayesian (BIC) information criterion were compared for each model, with the lower information criterion indicating a better fit.^18^ All analyses were conducted using STATA 16.^19^

### Patients

There was public involvement in the development of our portfolio of research on pregnancy planning, now known as the P3 study. A new PPI (Patient and Public Invovlement) group has been established which will be involved in the discussion of how these results are taken forward. This group is comprised of women aged 20–45 from across the UK, 20% of whom are from non-white ethnicities and half of whom have been pregnant at least once.

## Results

During the cognitive interviews with 16 women, no issues or concerns were raised with any of the questions on the RAS and no changes were made. Of the 994 women who took part in this study, 845 had engaged in sex with a male partner in the past year and were eligible to complete the RAS.

As seen in table 1, most of the participants were white (89.2%), over the age of 30 (60.7%), married (54.1%) and employed (73.9%). The mean age was 30.8 years, with almost half fitting into the 30–39 age range. The women in this sample were also highly educated, with 73.3% possessing an undergraduate-level degree or above.

**Table 1:** Reproductive Autonomy Scale scores by sociodemographic characteristics of study participants, UK 2018

Characteristic	N	%	Subscale of Reproductive Autonomy Scale			Total average
			Decision making	Freedom from coercion	Communication	
	Total=826		Mean score (SD)			
Age group (years)						
15–19	64	7.89%	2.48 (0.41)	3.78 (0.48)	3.55 (0.52)	3.33 (0.36)
20–29	255	31.44%	2.51 (0.37)	3.87 (0.31)	3.60 (0.47)	3.38 (0.27)
30–39	401	49.45%	2.48 (0.33)	3.91 (0.26)	3.58 (0.44)	3.38 (0.23)
40–50	91	11.22%	2.49 (0.35)	3.89 (0.29)	3.54 (0.46)	3.37 (0.25)
Missing	15	1.82%				
Relationship status						
Married	447	54.12%	2.45 (0.33)	3.91 (0.25)	3.59 (0.43)	3.38 (0.23)
Relationship—cohabiting	172	20.82%	2.52 (0.35)	3.91 (0.28)	3.57 (0.49)	3.39 (0.26)
Relationship—not cohabiting	130	15.74%	2.50 (0.38)	3.83 (0.35)	3.66 (0.37)	3.39 (0.26)
Single*	70	8.47%	2.69 (0.39)	3.77 (0.47)	3.34 (0.57)	3.30 (0.37)
Other	4	0.48%	2.44 (0.31)	3.70 (0.60)	3.50 (0.66)	3.27 (0.43)
Missing	3	0.36%				
Ethnicity						
White	737	89.23%	2.49 (0.35)	3.89 (0.29)	3.58 (0.44)	3.38 (0.25)
Mixed/multiple groups	20	2.42%	2.70 (0.32)	3.85 (0.32)	3.40 (0.70)	3.36 (0.33)
Asian/Asian British	50	6.05%	2.46 (0.35)	3.80 (0.43)	3.54 (0.48)	3.32 (0.29)
Black/African/Caribbean	10	1.21%	2.63 (0.21)	3.76 (0.37)	3.64 (0.40)	3.39 (0.26)
Other ethnic group	5	0.61%	2.45 (0.33)	3.76 (0.35)	3.56 (0.64)	3.31 (0.38)
Missing	4	0.44%				
Religion						
No religion	517	62.59%	2.51 (0.36)	3.90 (0.28)	3.58 (0.46)	3.39 (0.25)
Christian	239	28.93%	2.45 (0.34)	3.87 (0.31)	3.58 (0.46)	3.36 (0.26)
Other†	70	8.47%	2.47 (0.38)	3.80 (0.40)	3.56 (0.44)	3.34 (0.30)
Highest level of education						
Secondary school	50	6.05%	2.51 (0.41)	3.88 (0.38)	3.54 (0.62)	3.37 (0.35)
Further education‡	155	18.77%	2.52 (0.37)	3.82 (0.37)	3.56 (0.45)	3.36 (0.28)
Undergraduate degree	317	38.38%	2.49 (0.35)	3.89 (0.28)	3.59 (0.41)	3.38 (0.24)
Postgraduate degree	288	34.87%	2.48 (0.33)	3.91 (0.26)	3.58 (0.47)	3.39 (0.24)
Other	8	0.97%	2.34 (0.30)	3.68 (0.52)	3.40 (0.60)	3.20 (0.43)
Missing	8	0.97%				
Employment						
Employed	610	73.85%	2.49 (0.34)	3.90 (0.28)	3.58 (0.43)	3.38 (0.24)
Unemployed§	46	5.57%	2.53 (0.39)	3.93 (0.19)	3.57 (0.49)	3.36 (0.31)
Student	159	19.25%	2.42 (0.36)	3.83 (0.38)	3.58 (0.55)	3.37 (0.22)
Other	6	0.73%	2.67 (0.38)	3.8 (0.22)	3.40 (0.72)	3.33 (0.30)
Missing	5	0.61%				

*Women who were previously married/divorced were categorised based on their relationship status at the time of survey.

†Other religions included Buddhist, Hindu, Jewish, Muslim, Sikh, Arab and other.

‡Further education included A Level/AS/Highers/IB, college, diploma in higher education or equivalents.

§Unemployment included women who identified as full time mothers/housewives.

### Acceptability

Of the 845 eligible women, 826 (97.7%) completed all 14 items.

### Endorsement and targeting

The Decision Making subscale showed a satisfactory level of endorsement, with no response options exceeding 80%. In the Freedom from Coercion subscale, all five questions generated over 80% of responses in the ‘strongly disagree’ category. The Communication subscale showed a good range of responses, with all but one question option receiving response rates below the accepted 80%.

Total average reproductive autonomy scores for this sample were distributed between 1.5 and 3.7 (possible range 1–3.7) with a mean of 3.38 and a median of 3.43 (IQR 3.29–3.57). The scores for each subscale are shown in table 2.

**Table 2:** Item responses to the Reproductive Autonomy Scale by a sample of reproductive age, sexually active women (n=826), UK 2018

Decision making subscale		My partner	Both me and my partner	Me	
1	Who has the MOST say about whether you use a method to prevent pregnancy?	8 (1%)	408 (49.4%)	410 (49.6%)	
2	Who has the MOST say about which method you would use to prevent pregnancy?	13 (1.6%)	204 (24.7%)	609 (73.7%)	
3	Who has the MOST say about when you have a baby in your life?	33 (4%)	517 (62.6%)	276 (33.4%)	
4	If you became pregnant but it was unplanned, who would have the MOST say about whether you would raise the child, seek adoptive parents, or have an abortion?	15 (1.8%)	409 (49.5%)	402 (48.7%)	
**Freedom from Coercion subscale**		**Strongly disagree**	**Disagree**	**Agree**	**Strongly agree**
5	My partner has stopped me from using a method to prevent pregnancy when I wanted to use one	735 (89%)	68 (8.2%)	17 (2.1%)	6 (0.7%)
6	My partner has messed with or made it difficult to use a method to prevent pregnancy when I wanted to use one	747 (90.4%)	62 (7.5%)	12 (1.5%)	5 (0.6%)
7	My partner has made me use a method to prevent pregnancy when I did not want to use one	729 (88.3%)	76 (9.2%)	16 (1.9%)	5 (0.6%)
8	If I wanted to use a method to prevent pregnancy my partner would stop me	763 (92.4%)	54 (6.5%)	8 (1%)	1 (0.1%)
9	My partner has pressured me to become pregnant	766 (92.7%)	53 (6.4%)	4 (0.5%)	3 (0.4%)
**Communication subscale**		**Strongly disagree**	**Disagree**	**Agree**	**Strongly agree**
10	My partner would support me if I wanted to use a method to prevent pregnancy	24 (2.9%)	6 (0.7%)	132 (16%)	664% (80.4%)
11	It is easy to talk about sex with my partner	18 (2.2%)	86 (10.4%)	280 (33.9%)	442 (53.5%)
12	If I didn't want to have sex I could tell my partner	11 (1.3%)	23 (2.8%)	242 (29.3%)	550 (66.6%)
13	If I was worried about being pregnant or not being pregnant I could talk to my partner about it	10 (1.2%)	35 (4.2%)	235 (28.5%)	546 (66.1%)
14	If I really did not want to become pregnant I could get my partner to agree with me	13 (1.6%)	36 (4.4%)	263 (31.8%)	514 (62.2%)

### Reliability

The overall Cronbach’s α for the RAS was 0.75. The Freedom from Coercion subscale (0.81) and the Communication subscale (0.73) were both above 0.7; however, the Cronbach’s α for the Decision Making subscale was 0.61, as shown in table 3.

**Table 3:** Cronbach’s α scores for the Reproductive Autonomy Scale overall and by subscale

	Cronbach’s α
Reproductive Autonomy Scale	0.75
Subscale	
Decision Making	0.61
Freedom from Coercion	0.82
Communication	0.73

For test-retest reliability, the correlation coefficient between the baseline and 3-month total scores was 0.67. The correlation coefficient was 0.76 for the Decision Making subscale, 0.73 for the Communication subscale and 0.62 for the Freedom from Coercion subscale.

### Construct validity: hypothesis testing

Of the 826 women who completed all the items, 624 (75.5%) had used a method of contraception in the last 30 days and 200 (24.2%) had not. Of these 200, 63 (31.5%) were trying to get pregnant and 137 (68.5%) were trying to avoid pregnancy; 761 women were classed as trying to avoid pregnancy and were included for hypothesis testing. The Kruskal-Wallis test found a significant difference (p=0.013) in levels of reproductive autonomy between women who used contraception and those who did not. When adjusted for age, relationship status, ethnicity, education and employment, logistic regression analysis showed that for every one unit increase in total reproductive autonomy score, women had 2.9 times the odds of practising contraception (95% CI 1.58 to 5.33), confirming our hypothesis.

### Structural validity

Structural validity testing found the three-factor solution demonstrated a lower information criterion (AIC 12662.31; BIC 12921.73) than the one-factor solution (AIC 13220.63; BIC 13456.89) and is, therefore, a better fit. This confirms the three-factor structure of the RAS, consistent with the findings of the original development study, and suggests the RAS has good structural validity. The factor loadings for each item and the covariance between factors can be seen in table 4.

**Table 4:** Reproductive Autonomy Scale scores by subscale among a sample of reproductive age, sexually active women (n=826), UK 2018

Factor (subscale)	Mean	SD	Distribution	Possible range
Decision Making	2.49	0.35	1–3	1–3
Freedom from Coercion	3.88	0.3	1.6–4	1–4
Communication	3.58	0.45	1–4	1–4
Total average	3.38	0.26	1.5–3.7	1–3.7

## Discussion

The RAS was found to be both reliable and valid among a UK population of women, with satisfactory levels of endorsement and targeting. In terms of internal consistency, the reliability of the overall RAS, Communication, and Freedom from Coercion subscales were good. In terms of stability, the test-retest reliability of the RAS was fair-good. However, 3 months is longer than a standard test-retest period, which may have had a negative impact on the reliability due to genuine changes in women’s level of reproductive autonomy during that time. Our analysis confirmed the structural validity of the RAS’s three-factor structure. This analysis was a strength of our research because previous evaluations of the RAS did not attempt to confirm the structural validity of the scale.^8 9^

The RAS was found to be highly acceptable, with most of our sample understanding and completing all items without issue. There was a range of responses across the Decision Making and Communication subscales. However, responses on the Freedom from Coercion subscale indicate our sample was largely free from coercion. This is possibly because the women who were able to take part in this study were less likely to be vulnerable to coercion.

The construct validity of the RAS was demonstrated by the strong association between reproductive autonomy and contraceptive use among women who were trying to avoid pregnancy. Other evaluations of the RAS, including the original development study in the USA and studies in Vietnam^8^ and Brazil,^9^ chose to analyse reproductive autonomy in relation to recent instances of unprotected sex. Although unprotected sex and contraceptive use are closely related concepts, they are not interchangeable. There are multiple reasons why someone with high reproductive autonomy may choose to engage in unprotected sex, such as spontaneity or dissatisfaction with contraceptive methods.^20^ However, someone with very low reproductive autonomy is potentially less likely to access contraception due to wider structural and interpersonal limitations on their autonomy. Therefore, contraceptive use is arguably a more accurate indicator when attempting to identify those with low reproductive autonomy. However, another study testing the RAS among a sample of women in rural Brazil found that instances of unprotected sex did not correlate with low reproductive autonomy scores.^21^ This is likely due to the prevalence of unprotected sex between couples within this population.

The Decision Making subscale presented an interesting conceptual conundrum because women who report more shared decision making score lower on the RAS than women who report making the decision on their own, thus appearing to have lower reproductive autonomy. Data from the original development study and our UK evaluation both suggest that decisions about pregnancy outcomes are often considered a shared responsibility between a woman and her partner, yet women are expected to take primary responsibility in preventing pregnancy.^7^ The ‘contraceptive burden’ refers to the physical, mental and emotional burden placed on women as they are forced to take most of the responsibility for reproductive outcomes through the navigation and use of contraceptive methods.^22^ Within a relationship, shared responsibility for decision making may relieve some of this burden and not pose a threat to reproductive autonomy as suggested by the RAS.

It is also important to acknowledge the choices presented to women when trying to prevent pregnancy; they are often forced to navigate between the side effects of contraceptives and the risk of pregnancy.^22^ Future research should consider whether functioning within this paradox of choice can truly be considered autonomy. We should also consider the impact of distal factors, such as the provider landscape, since even women with high reproductive autonomy may struggle to access adequate contraceptive services in their local area.

While concepts of responsibility, choice and autonomy provide an interesting area of discussion and future research, shared decision making and lack of contraceptive choice do not threaten the utility of the RAS. Women who share reproductive decisions and use some form of contraception still retain a significant level of reproductive autonomy in their ability to contribute to reproductive outcomes.^7^ The main clinical utility of the RAS, like patient satisfaction measures, may be in identifying women who have the lowest levels of reproductive autonomy. In this case, a slightly lower score due to shared decision making does not threaten the value of the scale.

The RAS was originally developed to be utilised through the individual subscales, rather than providing an overall total average score for reproductive autonomy.^7^ As such, the original development paper provides no information on the total range or distribution of average scores. However, we decided to include total average scores as a useful way of estimating overall levels of reproductive autonomy among larger groups and identifying those at risk. The evaluation in Brazil also attempted to do this, but there is a lack of clarity in how they calculated their total average score as they state that possible scores range 1.6–3.57^9^ rather than 1–3.7, as calculated based on the number and ranges of subscale items ((4*3)+(5*4)+(5*4))/14=3.7. Despite this inconsistency, we believe the RAS holds value in future research both as an overall estimation of reproductive autonomy and a more in-depth tool to identify specific areas where autonomy is threatened.

Our sample did not generate the full range of reproductive autonomy scores, with 1.5–3.7 of a possible 1–3.7. This means women who had the very lowest reproductive autonomy did not take part in our study, potentially because those with higher reproductive autonomy were less likely to be marginalised and able to take part. Therefore, we recommend further exploration of this group in future studies. Further research may focus on testing the scale among gender-diverse people and could also directly explore the relationship between shared decision making and the contraceptive burden.

## Conclusion

The RAS is valid for use in the UK. Reproductive autonomy is a non-negotiable human right and a vital component of women’s empowerment. The RAS highlights the multidimensional nature of reproductive autonomy as a concept and illustrates the importance of communication, decision-making capacity and freedom from coercion in shaping reproductive outcomes. A validated UK version of the RAS can be utilised as a screening tool in sexual and reproductive health clinics across the UK, providing a way to identify those at risk of low reproductive autonomy who would benefit from further support and inform discussions between healthcare providers and their patients around reproductive autonomy.

## Data Availability

We have now made the data availabe in the UCL depository. Please use the following statement10.5522/04/21864561.
